# Ceftriaxone-Induced Immune Hemolytic Anemia: In Vitro Reversal with Peptide Inhibitor of Complement C1 (PIC1)

**DOI:** 10.1155/2019/4105653

**Published:** 2019-01-30

**Authors:** Kenji M. Cunnion, Lisa M. Feagin, Michael F. Chicella, Cortney L. Kaszowski, Pamela S. Hair, Jessica Price, William C. Owen

**Affiliations:** ^1^Department of Pediatrics, Eastern Virginia Medical School, 700 West Olney Road, Norfolk, VA 23507, USA; ^2^Children's Specialty Group, 811 Redgate Avenue, Norfolk, VA 23507, USA; ^3^Children's Hospital of the King's Daughters, 601 Children's Lane, Norfolk, VA 23507, USA; ^4^Department of Microbiology and Molecular Cell Biology, Eastern Virginia Medical School, 700 West Olney Road, Norfolk, VA 23507-1696, USA; ^5^Department of Pharmacy, Children's Hospital of the King's Daughters, 601 Children's Lane, Norfolk, VA 23507, USA

## Abstract

We report a case of ceftriaxone-induced immune hemolytic anemia in a 10-year-old with chronic active Epstein–Barr virus disease and hemophagocytic lymphohistiocytosis. After chemotherapy, she became febrile and received ceftriaxone. She rapidly developed respiratory failure and anemia. Her direct antiglobulin test was positive for IgG and C3. To confirm this was ceftriaxone-induced complement-mediated hemolysis, we adapted the complement hemolysis using human erythrocytes (CHUHE) assay by adding exogenous ceftriaxone to the patient's serum which enhanced lysis of her erythrocytes. We confirmed that ceftriaxone initiated a classical complement pathway-mediated hemolysis by *in vitro* reversal with peptide inhibitor of complement C1 (PIC1).

## 1. Introduction

Ceftriaxone-induced immune hemolytic anemia (CIIHA) is a rare complication that can lead to shock, multiorgan dysfunction, and death. Ceftriaxone is the second most common agent to cause drug-induced hemolytic anemia [1]. The majority of documented cases of CIIHA have occurred in children [1]. Cases of CIIHA are primarily reported in patients with an underlying condition of sickle cell disease or HIV with fatalities in 30% of those published [2]. Because this is a type 2 hypersensitivity reaction, the disease process can progress rapidly after re-exposure [3]. Here, we report a case of CIIHA in a 10-year-old female with chronic active Epstein–Barr virus (EBV) disease and hemophagocytic lymphohistiocytosis (HLH).

CIIHA is mediated by anticeftriaxone antibodies that bind to circulating ceftriaxone creating immune complexes that initiate classical complement pathway activation, which lyses erythrocytes [4]. Anticeftriaxone antibodies develop in 12.5% of patients frequently exposed to ceftriaxone [5], but CIIAH is a very rare complication. Standard evaluation for suspected CIIAH includes a direct antiglobulin test (DAT) and evaluation for the presence of anticeftriaxone antibodies. CIIAH is inferred as the diagnosis if the DAT is positive for complement and the presence of anticeftriaxone antibodies is confirmed. However, those test results do not prove that the anticeftriaxone antibodies are interacting with ceftriaxone to initiate classical complement pathway-mediated hemolysis.

In order to prove the mechanism of action for this patient, we utilize new technologies including the complement hemolysis using human erythrocytes (CHUHE) assay and peptide inhibitor of complement C1 (PIC1). The CHUHE assay utilizes human serum and human erythrocytes to measure complement-mediated hemolysis for the specific serum and specific erythrocytes that are coincubated [6, 7]. PIC1 is a small peptide inhibitor of classical pathway complement activation which acts by inhibiting enzymatic activation of C1, the first component of the cascade [8–10].

## 2. Methods

### 2.1. Ethics Statement

This case report was reviewed by the Eastern Virginia Medical School IRB and determined to not constitute human subjects research.

### 2.2. Reagents

The patient's blood and sera were provided as discarded deidentified samples from residual specimens in the blood bank. PIC1 derivative PA-dPEG24 [8] was synthesized by the PolyPeptide Group (San Diego, CA). Standard veronal complement buffers were utilized [6].

### 2.3. Modified CHUHE Assay

The patient's sera (0.1 ml) were combined with ceftriaxone (10 *μ*g/ml final concentration) in an ice-water bath for 30 minutes to enhance immune complex formation. This solution was then warmed to 24°C, and her erythrocytes (5 × 10^7^) were added, with or without PIC1 (final concentration 0.75 mM). Samples were incubated at 37°C for 1 hour, and hemolysis was stopped by the addition of 2.0 ml of GVBS-EDTA buffer (veronal-buffered saline with 0.1% gelatin and 10 mM EDTA). Erythrocytes were sedimented, and free hemoglobin was measured by spectrophotometry at 412 nm. Due to the limited amount of serum and erythrocytes available, we could only perform *n*=2 independent experiments performed in duplicate.

### 2.4. Statistical Methods

Quantitative data were analyzed determining means, standard error (SEM), and Student's *t*-test using Excel (Microsoft, Redmond, WA).

## 3. Case Presentation

A 10-year-old female with chronic active EBV disease and HLH was evaluated in the emergency department for fever and possible sepsis after recently receiving chemotherapy. In the emergency department, she received a dose of ceftriaxone (50 mg/kg). She had received ceftriaxone on three previous occasions with no history of adverse reaction. Within one hour, she developed back pain, tachycardia, and tachypnea. Over the next three hours, she developed worsening distress and failed continuous positive airway pressure support and required endotracheal intubation with mechanical ventilation. She also experienced hypotension requiring fluid resuscitation and a continuous epinephrine infusion.

Prior to receiving ceftriaxone, she had an erythrocyte hemoglobin concentration of 11.9 g/dL. Four hours later, her hemoglobin had decreased to 6.1 g/dL, followed by a point-of-care hemoglobin of 5.1 g/dL. There were spherocytes on her peripheral blood smear as well as red blood cell aggregation. A DAT report was sent after confirmation of the hemoglobin decrease and was positive for both IgG and C3. Urinalysis demonstrated hemoglobinuria and bilirubinuria. She required four packed red blood cell transfusions (each 10 mL/kg) over 72 hours, after which her hemoglobin stabilized at her initial baseline. High-dose methylprednisolone was begun during the first day of admission. On admission, one day later, and five days later, her total bilirubin levels were 1.5 mg/dL, 10.7 mg/dL, and 23.1 mg/dL, respectively, with 90% being unconjugated. On admission, her LDH was 514 U/L and increased to 42,093 U/L two days later. Her renal function declined 24 hours after ceftriaxone, with her BUN doubling from 12 mg/dL to 25 mg/dL and serum creatinine tripling from 0.3 mg/dL to 0.9 mg/dL. She continued to require inotropic blood pressure support for three days and required mechanical ventilation for sixteen days. Her presentation was highly suggestive of CIIHA and did not include red urine or severe anemia making the suspicion for cold agglutinin syndrome low.

**Figure 1:**
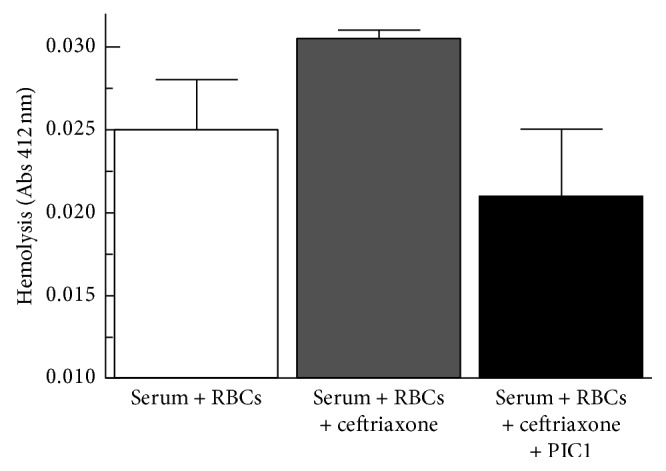
Modified CHUHE assay testing the patient's serum and erythrocytes. Addition of exogenous ceftriaxone increased hemolysis over baseline (*p*=0.02). Addition of PIC1 (0.75 mM) to the patient's serum, to which ceftriaxone was added, resulted in decreased hemolysis compared with the addition of ceftriaxone alone (*p*=0.03). Means ± SD are shown for 2 independent experiments.

The American Red Cross tested a blood sample from the day after admission and reported “Our testing was not definitive due to unexpected reactivity at the 37°C phase of testing with one set of controls. However, all controls were acceptable at the antihuman globulin phase of testing. The strong reactivity at 37°C and the reactivity at the antihuman globulin phase of testing indicate a strong likelihood that the patient has antibody to ceftriaxone.” In order to evaluate whether this was ceftriaxone-initiated hemolysis, we adapted the complement hemolysis using human erythrocytes (CHUHE) hemolytic assay utilizing the patient's serum and erythrocytes and adding exogenous ceftriaxone. These experiments showed that adding ceftriaxone (10 *μ*g/ml) to her serum increased complement-mediated hemolysis (*p*=0.02) of her erythrocytes (Figure 1). We also evaluated whether the hemolysis was occurring via the classical complement pathway by adding peptide inhibitor of complement C1 (PIC1). Adding PIC1 (0.75 mM) in addition to ceftriaxone to her serum and erythrocytes decreased hemolysis compared with adding ceftriaxone alone (*p*=0.03), returning hemolysis to the level of the no-ceftriaxone control. Together, these tests confirmed that the hemolysis was initiated by ceftriaxone and mediated via the classical complement pathway. These results raise the possibility that a classical pathway complement inhibitor could be employed therapeutically to moderate the disease process by blocking complement opsonization (e.g., C3b, iC3b, and C3d) of erythrocytes and inhibiting hemolysis. A terminal complement pathway inhibitor could also inhibit hemolysis, but would not stop complement opsonization of the erythrocytes.

## 4. Discussion

Here, we describe a case of CIIHA where we utilized new technologies to confirm the etiology and mechanisms of hemolysis. We modified the CHUHE assay [6, 7] by adding exogenous ceftriaxone and showed that hemolysis of the patient's erythrocytes was enhanced in the presence of ceftriaxone, confirming that ceftriaxone was the initiating stimulus. This result is consistent with a mechanism of “innocent bystander” complement-mediated hemolysis indicative of a drug-dependent IHA. We then added a classical complement pathway inhibitor, PIC1 [8], which inhibited ceftriaxone enhancement of hemolysis, demonstrating that the hemolysis was mediated by the classical complement pathway. These results also suggest the potential for complement inhibitors to be used clinically in treating CIIHA to prevent further hemolysis in the correct clinical setting with a C3-positive DAT. The long half-life of ceftriaxone of 8-9 hours [11] suggests that complement activation and hemolysis will continue to occur for a period of time as evident in this patient's course and can potentially be moderated.

## Abbreviations

EBV: Epstein–Barr virus
HLH: Hemophagocytic lymphohistiocytosis
DAT: Direct antiglobulin test
CHUHE: Complement hemolysis using human erythrocytes
PIC1: Peptide inhibitor of complement C1
CIIHA: Ceftriaxone-induced immune hemolytic anemia
HIV: Human immunodeficiency virus
LDH: Lactate dehydrogenase
BUN: Blood urea nitrogen
pRBC: Packed red blood cells.

## References

[1] Arndt P. A., Leger R. M., Garratty G. (2011). Serologic characteristics of ceftriaxone antibodies in 25 patients with drug-induced immune hemolytic anemia (CME). *Transfusion*.

[2] Neuman G., Boodhan S., Wurman I. (2015). Ceftriaxone-induced immune hemolytic anemia. *Annals of Pharmacotherapy*.

[3] Renard D., Rosselet A. (2017). Drug-induced hemolytic anemia: pharmacological aspects. *Transfusion Clinique et Biologique*.

[4] Kakaiya R., Cseri J., Smith S., Silberman S., Rubinas T. C., Hoffstadter A (2004). A case of acute hemolysis after ceftriaxone: immune complex mechanism demonstrated by flow cytometry. *Archives of Pathology & Laboratory Medicine*.

[5] Quillen K., Lane C., Hu E., Pelton S., Bateman S. (2008). Prevalence of ceftriaxone-induced red blood cell antibodies in pediatric patients with sickle cell disease and human immunodeficiency virus infection. *Pediatric Infectious Disease Journal*.

[6] Cunnion K. M., Hair P. S., Krishna N. K. (2016). Discriminating complement-mediated acute transfusion reaction for type O+ red blood cells transfused into a B+ recipient with the complement hemolysis using human erythrocytes (CHUHE) assay. *Transfusion*.

[7] Cunnion K. M., Hair P. S., Krishna N. K. (2016). Discriminating the hemolytic risk of blood type A plasmas using the complement hemolysis using human erythrocytes (CHUHE) assay. *Transfusion*.

[8] Sharp J. A., Hair P. S., Pallera H. K. (2015). Peptide inhibitor of complement C1 (PIC1) rapidly inhibits complement activation after intravascular injection in rats. *PLoS One*.

[9] Kumar P. S., Pallera H. K., Hair P. S. (2016). Peptide inhibitor of complement C1 modulates acute intravascular hemolysis of mismatched red blood cells in rats. *Transfusion*.

[10] Sharp J. A., Whitley P. H., Cunnion K. M. (2014). Peptide inhibitor of complement C1, a novel suppressor of classical pathway activation: mechanistic studies and clinical potential. *Frontiers in Immunology*.

[11] Zhao Y., Cudkowicz M. E., Shefner J. M. (2014). Systemic pharmacokinetics and cerebrospinal fluid uptake of intravenous ceftriaxone in patients with amyotrophic lateral sclerosis. *Journal of Clinical Pharmacology*.

